# Paradoxical Leadership and Involvement in Creative Task via Creative Self-Efficacy: A Moderated Mediation Role of Task Complexity

**DOI:** 10.3390/bs12100377

**Published:** 2022-10-02

**Authors:** Ki Baek Jung, Seung-Wan Kang, Suk Bong Choi

**Affiliations:** 1 College of Global Business, Korea University, Sejong City 30019, Korea; 2 College of Business, Gachon University, Seongnam 13120, Korea

**Keywords:** paradoxical leadership, creative self-efficacy, involvement in creative task, moderated mediation model

## Abstract

Modern organizational environments encounter serious competition and paradoxical situations. This study discusses the effect of paradoxical leadership on overcoming competitive and paradoxical situations happening in the Korean workplace. More specifically, it investigates the dynamic relationship between paradoxical leadership and involvement in creative tasks in a Korean context and examines whether creative self-efficacy positively mediates this relationship. Our research design addresses the moderating role and moderated mediating role of task complexity in the relationship between paradoxical leadership and the involvement in creative tasks via creative self-efficacy. The main hypotheses were tested by using a cross-sectional design and administering questionnaires to 268 employees working in Korean firms. Empirical analysis revealed that paradoxical leadership is positively related to involvement in creative tasks and creative self-efficacy and that creative self-efficacy positively mediated the relationship between paradoxical leadership and involvement in creative tasks. Importantly, as the relationship between paradoxical leadership and creative self-efficacy depends on task complexity, the mediated relationship was effective under high task complexity. Uncovering the relationship between paradoxical leadership and involvement in creative tasks with the mediating role of creative self-efficacy and the moderated mediating role of task complexity can provide useful theoretical and managerial implications.

## 1. Introduction

Modern organizations face competition. Consequently, organization managers often encounter organizational difficulties, for example, paradoxical situations where they pursue both efficiency and effectiveness, or short-term performance and long-term performance, or revenue and growth. In these environments, organizations need new leadership and organizational cultures to achieve the competitive advantage. One previous study has argued that leadership research focuses on specific leadership styles such as transformational, authentic, and servant leadership [1]. In addition, they found that empowering leadership and directive leadership have been actively studied. They pointed out that modern leadership theories have been heavily researched with transformational and charismatic leadership, whereas other leadership-style studies are being duplicated with similar concepts [1]. Thus, an integrated concept of leadership that encompasses modern leadership theory is required.

Paradoxical leadership has been researched in ambidexterity, paradox, dialectic, duality, and competitive value models, with the understanding that organizations must meet contradictory needs and employ new leadership styles to be competitive. Ambidextrous organization is defined as an organization that simultaneously achieves alignment and adaptability at the business-unit level [2], focusing on factors such as learning, innovation, and adaptability [3]. Previous studies have shown that ambidextrous organizations influence organization growth [4]. The organizational culture for building ambidextrous organizations presents various cultural characteristics. Organizations succeed when heterogeneous cultural characteristics appear at the same time [5]. Situational conditions affecting organizational culture include organizational goals, structures, management systems, social and cultural systems, and leadership styles [6].

However, ambidexterity and paradoxes have been mainly studied at the organizational level with few variables [7]. Thus, this study adopts an individual-level approach to test paradoxical conditions. Additionally, studies of ambivalence and paradoxes have rarely studied the behavior and leadership of paradoxical leaders, except those that deal with the actions or roles of competitive leaders [8,9,10]. Another study has outlined ways to realize ambidextrous organizational, team, and individual levels, but the content is limited to intellectual capital such as organizational, social, and human capital [11]. Traditionally, leadership studies have suggested that leadership styles affect the formation and maintenance of an organizational culture [12]. In other words, prior studies have not provided an integrated view of leadership in building an ambidextrous organizational culture and structure. Thus, we examine paradoxical leadership’s effectiveness in building an ambidextrous organization. 

Creativity has been defined as the development of novel and potentially useful ideas [13,14], and it is important for innovation and competitive advantage [15,16]. Traditionally, creativity studies have discussed outcomes of creative processes [17,18,19], but individual motivational viewpoints have been insufficiently investigated [20,21]. Nevertheless, the employee’s involvement in the task and active attitude toward creativity are important antecedents of performance. In this study, which was developed by compiling research on creativity and task commitment, we focused on involvement in the creative task. Involvement in creative tasks refers to the degree of engagement in which time and effort are used to perform one’s duties creatively [22]. When involvement in creative tasks is high, individuals will make efforts to apply new and useful ideas in their jobs to produce creative outcomes [23], which positively impacts the organization’s performance. Therefore, we aim to determine the impact of paradoxical leadership on involvement in creative task. 

Further, creative self-efficacy was expected to play a mediating role between paradoxical leadership and involvement in creative task. Creative self-efficacy is defined as the belief that individuals can produce creative results [19]. The idea of creative self-efficacy was formulated following a discussion of self-efficacy and creativity, the former being a key motivational driver of individual creativity [24,25]. Creative self-efficacy is an important factor in developing employee creativity [19,26,27,28,29], and it depends on factors such as personal duties, education, and leadership behavior [19]. For example, previous studies have suggested that both the creative role identity of employees and the expectations of leaders increase creative self-efficacy [30,31,32]. However, excessive demands for creativity have been shown to reduce creative self-efficacy and creativity. In this context, we expected that paradoxical leadership could affect creative self-efficacy and that creative self-efficacy could affect involvement in the creative task. Therefore, we expected that creative self-efficacy could play a mediating role between paradoxical leadership and involvement in the creative task.

Lastly, we assumed conditions of the organizational environment as a moderating variable in relation to tasks. Modern organizations have complex and systematic organizational structures to prepare for a competitive environment. Employee task structure and complexity can affect performance. Task complexity is defined as the level of stimulating and challenging demands related to a task [33]. Complex tasks require more proactive activities and explorations because complex tasks include uncertainty about how to complete the task [34], and they also require resources such as employee knowledge and skills [35,36]. These complex tasks are challenging and important, which can lead to positive attitudes and proactive motivation for tasks [14]. In addition, employees need meetings, feedback, leadership, or group coordination to address the difficulties and uncertainties of complex tasks [37]. In other words, a leader’s behavior and role could affect employees’ psychological states and performance when employees have complex tasks. Therefore, the extent of task complexity can vary the results of paradoxical leadership. Thus, we examined the moderating effect of task complexity on the relationship between paradoxical leadership and creative self-efficacy. We also investigated the moderated mediating role of task complexity in the relationship between paradoxical leadership and involvement in creative tasks via creative self-efficacy.

## 2. Theoretical Background and Hypotheses

### 2.1. Paradoxical Leadership and Involvement in Creative Task

Organizational ambidexterity can be defined as organizational structures, cultures, characteristics, and abilities that pursue two contradictory attributes simultaneously. These contradictory characteristics have been variously defined: exploration and exploitation [34], flexibility and efficiency [38], and incremental innovation and radical innovation [39]. Previous studies related to paradox have defined this as ‘seeking two mutually contradictory attributes at the same time’. In this context, conditions affecting organizational culture are suggested with a leadership style [6]. Leadership often influences the organizational culture [12]. Previous studies have also noted the importance of the leadership role in achieving organizational ambidexterity [7,40]. Paradoxical leadership was suggested as a new leadership style that can respond to the competitive environment and complex organizational structure faced by modern organizational environments [41]. 

Paradoxical leadership refers to leaders’ behavior to meet structural and employee needs simultaneously [41]. Zhang et al. [41] suggested five key features of paradoxical leader behaviors which are distinct from other existing types of leadership. First, paradoxical leaders are able to harmonize self-centeredness with other-centeredness. For example, they can maintain their central influence, while simultaneously sharing leadership with employees. Second, paradoxical leaders maintain both distance and closeness such as assigning vertical structural relationships from status, rank, and authority, while simultaneously trying to minimize status distinctions from employees’ demands [42]. Third, paradoxical leaders treat employees uniformly, while enabling individualization. They can use the value of uniformity or consistency [43], while simultaneously proceeding with individualization to overcome the problem of uniformity. Fourth, paradoxical leaders enforce the requirements of the work while allowing flexibility. Fifth, paradoxical leader maintains decision control, while allowing autonomy. For example, they control the decision-making process but provide employees with autonomy and empowerment in the process of completing the given task.

In this context, paradoxical leadership has some similarities and differences with other types of leadership. For instance, Fiedler’s contingency theory suggested effective leadership styles into task-oriented leadership and relation-oriented leadership according to the score of Least Preferred Coworker (LPC) based on situational factors such as leader–member relationship, task structure, and position of power [44]. Fiddler argued that an alternative leadership style is only effective depending on these situational factors. In contrast to Fiddler’s contingency theory, paradoxical leadership shows both leadership styles simultaneously to utilize the value of task-oriented leadership and relation-oriented leadership with effective consideration of mixed and complicated business situations today. 

Paradoxical leadership also has similarities with transformational leadership as a leadership style for enhancing creativity and innovation. Transformational leadership has suggested treating employees uniquely and personally, such as personalized considerations, because uniformity deprives them of their unique personal identity and talent. [45,46]. This characteristic of individual considerations can have a positive effect on creativity by stimulating proactive behavior and intrinsic motivation in employees. Paradoxical leadership, on the other hand, creates vertical structural relationships from status and authority and exerting uniform treatment of employees for control, while simultaneously maintaining personalized consideration, autonomy, and trying to minimize differences in status to employees’ needs. Therefore, it has a more integrated form of leadership by sharing some of the positive characteristics of transformational leadership for innovation and creativity improvement. Paradoxical leadership also represents a leader’s behavioral style that can effectively respond to all situations to improve employees’ creative process and performance.

Creativity, an antecedent of innovation, is being studied extensively [47,48,49]. However, to achieve sustainable growth and innovation, detailed consideration must be given to the individual level, such as an individual employee’s involvement in the creative task. Thus, we focused on involvement in creative tasks, which is the individual-level process for creative performance. Involvement in creative tasks refers to the degree of engagement in which time and effort are used to perform individuals’ duties creatively [22]. Involvement in creative tasks is the engagement of employees in creative processes, such as the creation of new and useful ideas with personal time and effort [22], which implies a subjective assessment of whether employees will concentrate on their work in a creative way. 

This study anticipated that paradoxical leadership would positively impact involvement in creative tasks for the following reasons. First, leadership is a key factor affecting creativity and innovation, and leaders contribute to employee creativity in a variety of ways [50]. Leaders can provide information, time, money, and creative efforts to enable employees to demonstrate creativity [51]. In addition, leaders’ individual consideration and relational support facilitate employees’ creative behavior [52]. Furthermore, leaders can make a creative climate and culture which influence employee creativity [53]. Paradoxical leadership behavior that combines control and autonomy to support creativity is effective in promoting creativity because this behavioral style makes employees perceive both tension and autonomy. 

Second, paradoxical leaders stay close to employees and share power but keep a proper distance [41]. A sense of distance from a leader can make employees feel in control. When employees are empowered, they put more effort into their job, especially with regard to the creative process which requires authority and responsibility. Third, paradoxical leaders seek effective solutions to maintain balance and harmony in a contradictory environment [41]. The paradoxical leader respects organizational requirements and individual flexibility simultaneously, which can instill dignity and confidence by recognizing individuality and demonstrating individual consideration, unlike task-oriented leaders [41]. As a result, paradoxical leadership positively impacts proficient, adaptive, and proactive behavior through a process that gives fresh inspiration to employees [41]. Proactive behavior is when individuals take proactive actions to predict or initiate changes in their task [54] including proactive and change-oriented behavior [55]. Proactive people tend to show enterprising behavior and more creativity when performing their job [56]. Paradoxical leadership affects both proactive behavior and task commitment for involvement in creative tasks. Thus, paradoxical leadership emphasizes the importance of tasks, but it also protects individual dignity, allowing employees to be confident and proactive. Employees’ creative behavior requires challenges, enthusiasm, and inner capability. Creative behavior only occurs when the motivation exists to do one’s task on one’s own [17]. When employees have a high level of intrinsic motivation, they can achieve stronger results [57]. Paradoxical leadership behavior in which leaders seek organizational performance while allowing flexibility can allow employees to set expectations for creative performance. As a result, paradoxical leadership increases intrinsic motivation for involvement in creative tasks. Thus, we hypothesized the following:

**H1.**  *Paradoxical leadership is positively related to involvement in creative tasks*.

### 2.2. Creative Self-Efficacy as a Mediator 

Creative self-efficacy refers to a person’s confidence in whether they can achieve creative results, such as completing challenging tasks and achieving goals creatively [19]. The concept of creative self-efficacy evolved from the concept of self-efficacy [19]. The leader’s role is important in determining employee self-efficacy [24]. Nevertheless, previous studies have dealt with task complexity, task-related self-efficacy, organizational hierarchy structure, tenure, and education levels as the antecedent of self-efficacy [17,19,52,58]. Therefore, a study of creative self-efficacy will need to shed light on the leader’s role as an antecedent of creative self-efficacy and the results of creative self-efficacy.

Paradoxical leadership aims to fulfill organizational and individual needs, in which effort has a positive effect on employee behavior and attitudes. Previous studies have shown that creative self-efficacy is a prerequisite for creativity [59,60] and employee creative performance [61]. Therefore, creative self-efficacy encourages employee creativity, both directly and indirectly. Moreover, employee creativity requires finding new solutions. Therefore, creative self-efficacy can help employees maintain awareness of existing problems and upcoming challenges. Thus, we assumed that creative self-efficacy positively mediates the relationship between paradoxical leadership and involvement in creative task in the following ways. 

Paradoxical leadership behavior of maintaining both distance from and closeness to employees keeps employees at a distance and distinguishes them from their bosses but is also perceived as friendly and not authoritarian [41]. This leadership behavior can make employees feel distant and charismatic [62], while paradoxical leaders enhance the level of leader-member exchange (LMX) by maintaining a sense of closeness. A charismatic leader improves employee efficacy so that employees perform well [63]. As a result, employees who embrace charismatic leadership also report positive job satisfaction and commitment [64]. Previous studies have confirmed that charismatic leadership affects self-efficacy, and self-efficacy affects job performance [65]. At the same time, a paradoxical leader expresses a sense of closeness with employees. Paradoxical leaders also allow mistakes in tasks through individual consideration [41]. As a result of this close relationship, an employee-leader trust relationship is established, and LMX quality improves. In other words, the leader supports the employee’s needs, emotions, and feedback, thereby improving the employee’s self-efficacy and interest in the job [66,67]. High-level relationships between employees and leaders enable employees to increase positive emotions, thinking, and behavior, as well as strengthen the confidence underlying creativity [68]. Therefore, paradoxical leadership that simultaneously demonstrates the strengths of LMX and charismatic leadership will give employees a strong sense of creative self-efficacy for creative tasks. This confidence is expected to help employees perform their duties creatively. 

The paradoxical leader also treats subordinates uniformly while allowing individual identity. Paradoxical leaders treat employees equally and distribute workload equally, but they also assign work in consideration of personal characteristics. This individual consideration is a pattern of behavior that can be seen in transformational leadership [45], unlike alternative leadership styles. Leadership styles play an important role in promoting job performance related to creativity [69,70]. Transformational leadership is likely to enable employees to exercise creative and independent thinking skills [71]. Individual consideration has a positive effect on employee creative self-efficacy [72]. Previous studies have identified the direct effects between transformational leadership and creative self-efficacy [73,74]. Traditionally, previous studies have argued that self-efficacy drives employees to perform their job with positive attitudes and thinking. In other words, employees with high levels of self-efficacy will be confident in their work. Therefore, employees with high creative self-efficacy make efforts to effectively cope with difficult situations by utilizing cognitive factors such as wide information exploration [19]. To be sure, creative work is difficult and challenging. A high level of self-efficacy could improve creative performance and provide solutions to employees’ creative problems [29]. In sum, a paradoxical leader’s “both–and choice” behavioral style gives employees a sense of self-efficacy in creative tasks, which promotes involvement in creative task. Thus, we hypothesized the following:

**H2.**  *Creative self-efficacy positively mediates the relationship between paradoxical leadership and involvement in creative task*.

### 2.3. Task Complexity as a Moderator

The definition of task complexity has historically been ambiguous and discussed from various perspectives. Campbell [75] classified task complexity as a characteristic of the task itself and later defined it further as an attribute that increases the task’s amount, variety, and rate of change in information [76]. In other words, high task complexity requires complex abilities from those who perform the tasks [77]. A more complex task requires a high degree of interdependence because of its increased ambiguity and non-routine, unstructured characteristics [78]. Task complexity has been known to affect employees’ psychological states and behaviors. Previous studies have confirmed that task complexity affects positive outcomes such as motivation and performance [79]. Task complexity is a challenging stimulus and induces a sense of accomplishment [80]. Thus, if task complexity is high, the employee performs a task that they perceive as challenging, and in this case, an employee who feels control due to paradoxical leadership but also has authority will have confidence in their competence. Therefore, higher task complexity strengthens competence, creates a positive attitude, and tends to yield the positive effects of paradoxical leadership.

According to previous studies [81,82], leadership effectiveness depends on certain conditions. Leadership has more impact on business processes and performance when leaders need to play more roles [82]. On the other hand, the preceding study on task complexity argued that high task complexity requires high interdependence [83]. Further, high task complexity increases the individual’s effort and motivation to learn [84]. Depending on the degree of task complexity, there is less need for leadership in a simple and routine task environment [85]. However, since high task complexity has characteristics such as task difficulty and diversity in task structure, a high level of cooperation and coordination is required for performance [86]. In other words, the demand for interaction between the leaders or members of the organization increases. In this context, the positive behavioral strategy of paradoxical leadership can increase the level of creative efficacy by inducing more challenging stimulation of employees through complex tasks. When the task is complex, it is not standardized. Therefore, high autonomy and discretion should be given to employees in the task process. Task complexity can act as a motivational factor for individuals [87]. On the other hand, a leadership intervening and effectively performing a task is important for complex tasks [88]. Paradoxical leaders treat employees equally and have individual autonomy through individual considerations. In other words, it will be possible to increase creative self-efficacy and performance by sharing essential resources for performance.

When task complexity is high, employees are motivated to pursue efficient strategies to successfully complete their task [66,89,90]. In discussing task complexity, indirect relationships or cognitive factors play a more important role than direct relationships [91]. Therefore, this paper focuses on individual perceived task complexity by employees. In other words, employees will recognize the need for individual interaction with leaders when there is high task complexity. In particular, paradoxical leadership behavior includes elements that positively impact creativity, such as shared leadership and power-sharing leadership, which was expected to be moderated depending on the level of task complexity. Thus, we hypothesized the following:

**H3.**  *Task complexity positively moderates the relationship between paradoxical leadership and creative self-efficacy*.

### 2.4. Integrated Model: A Moderated Mediating Effect

Our study was based on a previously hypothesized pattern of moderation implying moderated mediation, whereby a mediated effect varies as a function of a third variable [92]. When task complexity is high, the indirect effect of paradoxical leadership on involvement in creative tasks via creative self-efficacy is significantly enhanced, thereby strengthening the mediating role of creative self-efficacy in the relationship between paradoxical leadership and involvement in the creative task. In contrast, when task complexity is low, the positive effect of paradoxical leadership on creative self-efficacy is weaker, thereby also weakening the mediating role of creative self-efficacy in the relationship between paradoxical leadership and involvement in creative tasks. As discussed earlier, paradoxical leadership is an antecedent of involvement in creative tasks. The “both–and choice” behavioral style of paradoxical leadership not only puts employees under pressure to complete tasks but also guarantees autonomy. In addition, when employees experience equal treatment and individual consideration from paradoxical leaders, they may not only feel a sense of belonging to the organization but also a sense of respect. These characteristics can be positive in an organization that allows employees to work harder and commit to the organization. Thus, we hypothesized the following:

**H4.**  *The strength of the relationship between paradoxical leadership and involvement in creative task, mediated by creative self-efficacy, varies depending on the extent of task complexity, i.e., the indirect effect of paradoxical leadership on involvement in creative task via creative self-efficacy is stronger when task complexity is high*.

**Figure 1:**
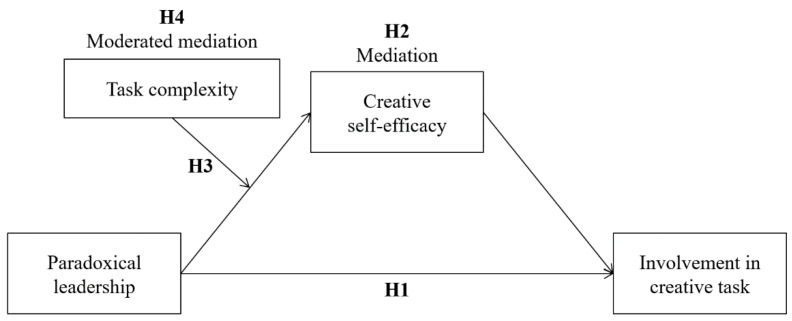
The theoretical research model.

The theoretical model of this study is depicted in Figure 1.

## 3. Methodology

### 3.1. Sample and Procedure

For empirical analysis, we adopted a non-random sampling method considering employee perception of key variables used in this study, such as paradoxical leadership and task complexity. We used a survey to obtain data from employees who work at Korean firms and collected data through online and offline questionnaires. We used this method because employees directly experience the influence of leadership. In addition, task complexity can be more affected by the employee’s perception of a task’s complexity than from an objective perspective on a task’s complexity. 

Following previous studies’ suggestions to use a non-probability method [93,94], we more effectively selected sample groups based on our judgment of and preference for their research objectives. We contacted Human Resources managers to explain the purpose of the survey and obtain their permission. We also tried to ensure anonymity and consider the diversity of sampling regarding gender, tenure, and age. Lastly, we assured that participants could quit the survey at any time, and then distributed the questionnaire. We collected 295 questionnaires and used 268. Respondent demographics were as follows. Among the 268 respondents, 54.9% were female, 83.2% were staff and 34.7% were service workers, and 26.5% were office workers. The average age of the respondents was 31.60 years (SD = 8.30), the average organizational tenure was 6.07 years (SD = 6.06), and the average duration of education was 15.67 years (SD = 1.66).

### 3.2. Measures

Respondents rated items on a five-point Likert-type scale from 1 (strongly disagree) to 5 (strongly agree). Following Brislin’s [95] translation and back-translation procedure, we asked a professional translator to develop a Korean version of the original English instrument, and this was then back-translated into English by a bilingual academic who had not seen the original English version. We asked this translator to comment on any ambiguously worded items, and he did not suggest any noteworthy changes.

#### 3.2.1. Paradoxical Leadership

Paradoxical leadership is defined as leadership that integrates conflicting situations in organizational management, simultaneously meeting organizational needs and employees’ personal needs [41]. We used the paradoxical leadership behavior questionnaire developed by Zhang et al. [41]. Sample items included: “My boss communicates with subordinates uniformly without discrimination but varies his or her communication styles depending on their individual characteristics or needs.”, “My boss has a high self-opinion, but shows awareness of personal imperfection and the value of other people.”, and “My boss makes decisions about big issues, but delegates lesser issues to subordinates.” The Cronbach’s alpha was 0.944.

#### 3.2.2. Involvement in Creative Tasks

Involvement in creative tasks is defined as an employee’s involvement in the creative process of investing time and effort to develop new and useful ideas. We used four items developed by Carmeli et al. [96]. Sample items included: “I try out new ideas and approaches to problems.” and “I identify opportunities for new products/processes.” The Cronbach’s alpha was 0.903.

#### 3.2.3. Creative Self-Efficacy

Creative self-efficacy refers to a person’s confidence in whether they can achieve creative results, such as completing challenging tasks and achieving goals creatively. We used three items developed by Tierney and Farmer [19]. Sample items included: “I often suggest new and innovative ideas.” and “I am confident that I can solve the problem creatively.” The Cronbach’s alpha was 0.903.

#### 3.2.4. Task Complexity 

Task complexity can be defined as an attribute that increases the task’s amount, variety, and rate of change in information. We used three items developed by Dean and Snell [97]. Sample items included: “How much technical knowledge do the jobs in this unit require?” and “How complicated are the jobs in this unit?” The Cronbach’s alpha was 0.869.

## 4. Results

### 4.1. Descriptive Statistics and Correlations

Correlation analysis was conducted to investigate the relationship and direction between the variables in this study. The means, standard deviations, reliabilities, and correlations among the key variables are shown in Table 1. Paradoxical leadership was related to involvement in creative task (r = 0.323, *p* < 0.001). Involvement in creative task was related to creative self-efficacy (r = 0.662, *p* < 0.001), and task complexity (r = 0.238, *p* < 0.001). In addition, we carried out the reliability to test that the measured data were suitable for empirical analysis prior to hypothesis testing. The Cronbach’s alpha exceeded 0.70, indicating that the reliability of the measurement tool is high [98].

### 4.2. Confirmatory Factor Analysis (CFA)

We conducted a confirmatory factor analysis (CFA) on the measures of the key variables to test factor structure and construct validity. We modeled four factors: paradoxical leadership, creative self-efficacy, task complexity, and involvement in creative tasks. The results are shown in Table 2. This theoretical four-factor model provided a reasonable fit to the data (χ^2^ = 388.908, df = 246, CFI = 0.970, TLI = 0.964, RMR = 0.039, RMSEA = 0.047). Chi-square difference tests revealed that the four-factor model fits the data significantly better than several alternative measurement models in Table 2. These results confirmed the theoretical four-factor model, thus supporting discriminant validity among the measures. In addition, the average variance extract (AVE) and composite reliability (CR) value of all the variables satisfied the criteria (AVE > 0.5, CR > 0.7), thus supporting the convergent validity and reliability of the constructs [99,100].

### 4.3. Hypotheses Test

We tested the Hypotheses 1–3 by hierarchical regression analysis and the results are presented in Table 3. We calculated interaction terms which needed to test the moderating effects of hypothesis 3. Before calculating the interaction term, we attempted to solve the problem of multicollinearity by performing a mean centering method [101]. In addition, the results carried out the value of the variance inflated index, the maximum VIF of the key variables is 1.355, so there is no multicollinearity problem.

Paradoxical leadership was positively related to involvement in the creative task (β = 0.291, *p* < 0.001) after controlling for gender, age, tenure, education, position, and job type in Model 5. Thus, hypothesis 1 was supported. To test hypothesis 2 regarding the mediating role of creative self-efficacy in the relationship between paradoxical leadership and involvement in creative tasks, we followed the procedure established by Baron and Kenny [102]. First, by testing hypothesis 1, we already confirmed the positive effect of paradoxical leadership on involvement in creative tasks. Second, Model 2 showed that the paradoxical leadership was positively associated with creative self-efficacy (β = 0.275, *p* < 0.001). Finally, in Model 6, creative self-efficacy was positively related to involvement in creative tasks (β = 0.589, *p* < 0.001), explaining significant additional variance in involvement in creative tasks (ΔR^2^ = 0.275, *p* < 0.001). The effect of paradoxical leadership on involvement in creative tasks became weaker but was still significant (β = 0.129, *p* < 0.01) indicating partial mediation. To confirm this result, we applied the Preacher and Hayes [103] indirect test, which applies the bootstrap method to obtain more reliable estimates. The bootstrap results confirmed a significant indirect effect (indirect effect = 0.18, SE = 0.06, 95% CI [0.12, 0.37]). Thus, hypothesis 2 was supported.

**Figure 2:**
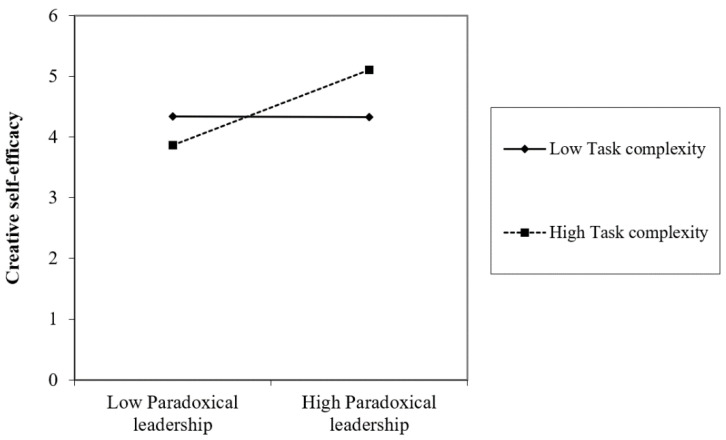
Moderating effect of task complexity on the relationship between paradoxical leadership and creative self-efficacy.

In hypothesis 3, we expected that task complexity moderates the relationship between paradoxical leadership and creative self-efficacy. Regarding the moderating role of task complexity, the interaction term of paradoxical leadership and task complexity significantly predicted creative self-efficacy (β = 0.220, *p* < 0.001; ΔR^2^ = 0.053, *p* < 0.001) in Model 3. In Figure 2, to facilitate the interpretation of the moderating effect, a graph is drawn that distinguishes between high and low task complexity groups based on the average value of task complexity. As shown in Figure 2, there was a difference in the relationship depending on the degree of task complexity. Thus, hypothesis 3 was supported.

To test hypothesis 4 regarding integrative moderated mediation, we examined whether the indirect effect of paradoxical leadership on involvement in creative task via creative self-efficacy was moderated by task complexity (i.e., conditional indirect effect). To test the conditional indirect effect, we utilized Hayes’ [104] PROCESS program. The indirect effect of paradoxical leadership on involvement in creative tasks via creative self-efficacy was estimated at high (+1SD) and low levels (−1SD) of task complexity with the bootstrap method. The analysis results are presented in Table 4. The results indicated that the indirect effect was significant for high task complexity (conditional indirect effect = 0.3140, SE = 0.0590, 95% CI [0.1999, 0.4287]) but was not significant for low task complexity (conditional indirect effect = 0.0508, SE = 0.0546, 95% CI [−0.0400, 0.1746]), thus supporting hypothesis 5.

## 5. Discussion and Implications

In the context of Korean employees, our study investigated the link between paradoxical leadership and involvement in creative tasks, the mediating role of creative self-efficacy in this relationship, and the moderating role of task complexity in the relationship between paradoxical leadership and creative self-efficacy. The results showed that paradoxical leadership positively impacts involvement in creative tasks and that creative self-efficacy not only mediated the relationship between paradoxical leadership and involvement in creative task, but also had a moderating effect on task complexity in the relationship between paradoxical leadership and creative self-efficacy. Lastly, we found that task complexity moderates creative self-efficacy’s indirect effect on the relationship between paradoxical leadership and involvement in creative tasks.

Our study has three theoretical implications. First, our study confirms the effectiveness of the paradoxical leadership in Korean firms. Previous leadership theories focused on the unique characteristics of a leader or tested the behavioral patterns of a leader [105,106]. According to contingency theory, leadership characteristics change depending on the situation and analyze the type of leadership suitable for various situations [107,108]. Our study has confirmed the effectiveness of paradoxical leadership in the competitive and contradictory situations of modern society by deviating from these existing theories. This suggests that leadership needs to consider and harmonize both organizations and individuals.

Second, our study empirically confirms that creative self-efficacy mediates paradoxical leadership and involvement in creative task. Although prior studies have analyzed creative self-efficacy, creativity, creative performance, and involvement in creative task [22,27,73], or have been interested in the outcomes of creative processes [17,18,19], few studies have studied creativity’s direct relationships with or indirect effects on leadership. This study contributes to prior creativity research by identifying the mechanism of creativity, which depends on individual motivation and psychological state, in relation to paradoxical leadership. Therefore, our research extends the application of paradoxical leadership to understanding the creative process.

Third, our findings have shown that the positive relationship between paradoxical leadership and creative self-efficacy becomes stronger as task complexity increases, confirming task complexity’s moderated mediating effect. Prior studies on task complexity have shown that there is an important relationship between task complexity and individual creativity [14]. Our study expands the scope of previous research by proving the relationship between paradoxical leadership and individual creative self-efficacy. These findings demonstrate the effectiveness of paradoxical leadership while also showing that it can strengthen or weaken the effectiveness of paradoxical leadership depending on the degree of task complexity.

Our results also suggest several managerial implications. First, our results provide implications for leadership education. With increasing theoretical interest in paradoxical situations since the late twentieth century, various studies have attempted to construct a theory about organizational paradoxical management [5,109,110]. In addition, managers must balance contradictions. Our findings suggest that managers need to consider both organizational and individual needs simultaneously. Managers encourage employees’ creative thinking by emphasizing both the importance of the task and individual autonomy. Therefore, companies could establish leadership development programs for managers to strengthen their leadership skills.

Second, we confirmed the process for creativity by checking the effect of creative self-efficacy. We provided an empirical basis for the need for making organizational efforts toward encouraging involvement in creative tasks. Creativity is an important factor in the survival of modern organizations [18,111]. Therefore, when an organization pursues creativity for innovation, it should design and implement programs to enhance the level of creative self-efficacy and involvement in creative tasks. For example, to avoid the fear of creative work, a new employee education program could be implemented to develop adaptability to challenging tasks and creative tasks. In addition, leaders could redesign evaluation systems by including whether to participate in the project or propose creative ideas.

Third, we provide implications for task design by confirming the moderating effect of task characteristics when paradoxical leadership has a positive impact on the creative process. When tasks are complex, the methods and processes required for task performance are not structured [112]. Therefore, tacit knowledge is likely to be applied to the task’s performance [113]. Contact with various peers and different layers is required to effectively manage task-related complexity. Our findings suggest that paradoxical leadership can have a positive effect on the creative process through close interactions with employees. For example, management may induce communication and information acquisition through horizontal structural design or implement mentoring programs in the face of high task-related uncertainty.

Our study has several limitations that should be considered in future research. First, the data was collected through a self-report questionnaire. Therefore, there is a possibility of a common-method-bias problem [114]. We tried to ensure anonymity in the data collection process to reduce this problem [114]. However, future research should separate the measurement source or collecting time to overcome the common method bias problem. Second, our data was cross-sectional based on a point in time. Therefore, we suggest that data should be collected longitudinally to improve the robustness of the results. Third, data collection and analysis were conducted on the individual level. However, team-level analysis is important. Therefore, it would be worth examining the effects of leadership and other variables that can be studied from the group or team level such as organizational climate, climate for innovation, and organizational culture.

## Figures and Tables

**Table 1:** Means, standard deviations, correlations, and reliabilities.

Variables		Mean	SD	1	2	3	4	5	6	7	8	9	10
1	Gender	0.55	0.50										
2	Age	31.60	8.30	−0.010									
3	Tenure	6.07	6.06	0.127 *	0.796 ***								
4	Position	1.31	0.82	−0.137 *	0.451 ***	0.497 ***							
5	Education	15.67	1.66	−0.098	0.033	0.025	0.146 *						
6	Job type	3.05	1.54	0.437 ***	0.028	0.101	−0.086	−0.064					
7	PL	3.53	0.65	−0.085	−0.048	−0.041	0.028	0.156 *	−0.002	(0.944)			
8	CSE	3.33	0.80	−0.308 ***	−0.162 **	−0.186 **	−0.002	0.034	−0.237 ***	0.297 ***	(0.903)		
9	TC	3.63	0.76	0.028	0.030	0.045	0.068	0.136 *	0.154 *	0.289 ***	0.119	(0.869)	
10	ICT	3.28	0.75	−0.309 ***	−0.089	−0.094	0.135 *	0.088	−0.167 **	0.323 ***	0.662 ***	0.238 ***	(0.903)

Note. *N* = 268, * *p* < 0.05, ** *p* < 0.01, *** *p* < 0.001, the values in parentheses denote Cronbach’s alphas, PL: paradoxical leadership, CSE: creative self-efficacy, TC: task complexity, ICT: involvement in creative task.

**Table 2:** Model fit statistics for measurement models.

Model	χ^2^ (*df*)	CFI	TLI	RMR	RMSEA	Δ χ^2^ (df)
Theoretical four-factor model (PL, CSE, TC, ICT)	388.908 (246)	0.970	0.964	0.039	0.047	
Three-factor model (PL and TC, CSE, ICT)	751.334 (249)	0.896	0.875	0.059	0.087	362.426 (3) ***
Two-factor model (PL and CSE and TC, ICT)	1390.906 (252)	0.764	0.720	0.095	0.130	1001.998 (6) ***
One-factor model (PL and CSE and TC and ICT)	1743.530 (253)	0.692	0.634	0.103	0.149	1354.622 (7) ***

Note. PL: paradoxical leadership, CSE: creative self-efficacy, TC: task complexity, ICT: involvement in creative task, *** *p* < 0.001.

**Table 3:** Results of hierarchical multiple regression.

Variables	Creative Self-Efficacy			Involvement in Creative Task		
	Model 1	Model 2	Model 3	Model 4	Model 5	Model 6
Gender	−0.244 ***	−0.220 **	−0.237 ***	−0.269 ***	−0.243 ***	−0.114 *
Age	−0.136	−0.116	−0.128	−0.152	−0.131	−0.063
Tenure	−0.057	−0.058	−0.042	−0.021	−0.022	0.012
Position	0.044	0.036	0.014	0.169 *	0.160 *	0.139 *
Education	0.002	−0.039	−0.041	0.041	−0.002	0.021
Job type	−0.117	−0.131 *	−0.119	−0.026	−0.040	0.037
PL		0.275 ***	0.272 ***		0.291 ***	0.129 **
TC			0.073			
CSE						0.589 ***
PL×TC			0.220 ***			
R^2^	0.135	0.209	0.262	0.129	0.211	0.486
ΔR^2^		0.074 ***	0.053 ***		0.082 ***	0.275 ***

Note. *N* = 268, PL: paradoxical leadership, TC: task complexity, CSE: creative self-efficacy. Standardized coefficients are reported, * p < 0.05, ** p < 0.01, *** p < 0.001.

**Table 4:** Moderated mediation results for conditional indirect effect.

Task Complexity	Boot Indirect	Boot SE	95% of Confidence Intervals	
	Effect (β)		*Boot* LLCI	*Boot* ULCI
*M* −1SD	0.0508	0.0546	−0.0400	0.1746
*Mean*	0.1824	0.0414	0.1060	0.2683
*M* +1SD	0.3140	0.0590	0.1999	0.4287

## Data Availability

Not applicable.
